# Allergy-Test-Based Elimination Diets for the Treatment of Eosinophilic Esophagitis: A Systematic Review of Their Efficacy

**DOI:** 10.3390/jcm11195631

**Published:** 2022-09-24

**Authors:** Constantinos Pitsios, Emilia Vassilopoulou, Katerina Pantavou, Ingrid Terreehorst, Anna Nowak-Wegzryn, Antonella Cianferoni, Georgios Panagiotis Tsigkrelis, Maria Papachristodoulou, Stefanos Bonovas, Georgios K. Nikolopoulos

**Affiliations:** 1 Medical School, University of Cyprus, Nicosia 1678, Cyprus; 2 Department of Nutritional Sciences and Dietetics, International Hellenic University, 57400 Thessaloniki, Greece; 3 ENT Department, Amsterdam University Medical Centre, 1105 Amsterdam, The Netherlands; 4 Allergy and Immunology, Department of Pediatrics, Hassenfeld Children’s Hospital, New York University Grossman School of Medicine, New York, NY 10016, USA; 5 Children’s Hospital of Philadelphia, Perelman School of Medicine, University of Pennsylvania, Philadelphia, PA 19104, USA; 6 Department of Biomedical Sciences, Humanitas University, 20090 Milan, Italy; 7 IRCCS Humanitas Research Hospital, 20089 Milan, Italy

**Keywords:** eosinophilic esophagitis, elimination diet, allergens, allergy skin tests, skin prick tests, specific IgE, atopy patch tests, specific IgG4, prick to prick test

## Abstract

Eosinophilic esophagitis (EoE) is an immune-mediated esophageal disorder, linked with sensitization to food and airborne allergens. Dietary manipulations are proposed for the management of EoE inflammation and are often successful, confirming the etiological role of food allergens. Three different dietary approaches are widely used: the elemental, the empirical, and the allergy-test-driven approach. We performed a systematic review to assess the evidence on the association of allergens, detected by allergy tests, with clinically confirmed triggers of EoE. We systematically searched PubMed, Scopus, Embase, and the Cochrane Library, through 1 June 2021. We sought studies examining the correlation of skin-prick tests (SPT), atopy patch tests (APT), specific IgE, and serum-specific IgG4, with confirmed triggers of EoE. Data on the use of prick–prick tests were also extracted. Evidence was independently screened by two authors against predefined eligibility criteria. Risk of bias was assessed with the ROBINS-I tool. Of 52 potentially eligible studies, 16 studies fulfilling quality criteria were included. These studies used one to three different allergy tests detecting food sensitization. The positive predictive value was generally low to moderate but higher when a combination of tests was used than single-test evaluations. None of the selected studies used serum-specific IgG4. Although an extreme methodological variability was noticed in the studies, allergy-based elimination diets were estimated to be efficient in 66.7% of the cases. The efficacy of targeted elimination diets, guided by SPT, sIgE, and/or APT allergy tests, does not appear superior to empirical ones. In the future, tests using esophageal prick testing or ex vivo food antigen stimulation may prove more efficient to guide elimination diets.

## 1. Introduction

Eosinophilic esophagitis (EoE) is a chronic inflammatory disease with symptoms of esophageal dysfunction, similar to those of gastrointestinal reflux disease, characterized by marked eosinophil-predominant inflammatory infiltration of the esophagus [1,2]. The diagnosis is clinicopathologic, with 15 or more eosinophils per high-power field in any of multiple biopsy specimens obtained [1,3]. Symptoms of EoE vary with age [2]. In infants and pre-school children, vomiting, failure to thrive, and food refusal are common features, while in school-age children, reduced appetite, slow eating, difficulty in swallowing, and vomiting are the usual presenting symptoms [4,5]. The most common symptoms in puberty and adulthood are dysphagia, food impaction, heartburn, and chest pain [1,5].

Concomitant atopic diseases, including bronchial asthma, allergic rhinitis, and atopic dermatitis, are more frequently reported in patients with EoE than in the general population. In a meta-analysis, the relevant odds ratios ranged from 2.8 to 5.1 times greater, with no significant differences when children and adults were considered separately [6]. Patients with established food allergy are also considered to have a tendency for subsequent EoE development [7]. Although EoE is highly related to atopy, the underlying pathophysiology does not clearly involve allergen-specific IgE antibodies [8,9]. EoE seems instead to be a T-helper 2 (Th2) cell-mediated immune disorder correlated with sensitization to airborne and/or food allergens but not developing through an IgE-mediated mechanism [9]. Exposure to food antigens or aeroallergens trigger a specific immune response, likely T mediated, leading to local esophageal inflammation in a genetically predisposed individual [10,11,12].

The epithelium has a key role in instructing the immune system towards allergen sensitization instead of tolerance. Genetically predisposed individuals are more prone to the secretion of Th2-promoting cytokines (thymic stromal lymphopoietin, IL-25, IL-33) by epithelial cells and prostaglandin D_2_ (PGD_2_) by mastcells [8,13,14,15]. These events are followed by the stimulation of local Th2 cytokine (IL-4, IL-5, IL-13) and PGD_2_ release by CD4^+^ Th2 cells (termed pathogenic effector peTh2 cells), leading to the chemotaxis and activation of eosinophils and innate lymphoid cells ILC2. A further Th2 cytokine release causes a self-enhancing loop [9,16,17]. An idea, also connecting IgE-mediated allergy to EoE, suggests that IgG4 antibodies—known as neutralizing the IgE effects—are generated in atopic individuals, contributing to the pathogenesis of EoE [9]. Data suggesting a potential role of tissue-resident IgG4 are its staining in active EoE esophageal biopsies and the decrease of IgG4 levels in tissue biopsies, as well as in plasma after food elimination and in parallel with symptoms’ improvement [9,18].

A combination of proton pump inhibitors, topical glucocorticoids and/or food antigen avoidance is the first-line anti-inflammatory treatment of EoE [3,5,19]. Dietary interventions have confirmed the etiological role of food allergens in this eating disorder. Three different dietary approaches are practiced, each aiming to minimize the effect of dietary allergens on the esophageal mucosa: an elemental formula diet; “empiric” food-elimination diets (FED) based on most involved foods or food groups (e.g., two-, four-, and six-food diets); and, lastly, diets based on multimodality allergy testing [1]. The relative allergy tests detect specific IgE [with skin prick tests (SPT) or in serum (sIgE)], specific IgG4 or cell-mediated responses by means of atopy patch tests (APT) [7,20]. Although elimination diets offer a positive effect, they have clear disadvantages, including costs, the limitation of food options, and the risk of nutritional deficiencies.

Elimination diets can serve as the first step in identifying the culprit allergens in EoE; after symptom remission, the eliminated food allergens can be reintroduced sequentially and food triggers can be defined clinicopathologically [21]. In the present review, the outcomes of the food-reintroduction diagnostic approach served as a comparator to the results of allergy tests (SPT, sIgE, APT) that have been used in several studies as diagnostic tools of EoE food triggers. Our aim was to review the literature on the diagnostic value of allergy tests that can be performed in every day practice, namely SPT, APT, and sIgE in serum [22].

## 2. Materials and Methods

The detailed methods of our systematic review have been reported in the published protocol [22]. A succinct description of the employed methods follows.

### 2.1. Search Strategy and Selection Criteria

A sensitive search strategy was developed, and validated study-design filters were applied to investigate four electronic databases (PubMed, Scopus, Embase, and the Cochrane Library). Search terms included: *eosinophilic esophagitis*, *EoE*, *skin prick test*, *SPT*, *specific immunoglobulin E*, *specific IgE*, *sIgE*, *atopy patch test*, *APT*, *immunoglobulin G4*, and *IgG4*. Reference lists of the retrieved records were checked for additional potentially eligible studies [23]. The databases were searched from their inception to 1 June 2021.

This systematic review included studies involving patients with EoE of any age and examining the relationship between changes in esophageal mucosa’s histology triggering EoE symptoms and the results of allergy testing. Inclusion criteria are reported in Appendix A. Only studies with original data, without reference to duplicated data, were included. Studies using therapeutical procedures, other than diet elimination, were excluded. Exclusion data are reported in Table A1 and in the published protocol [22].

Search results were uploaded into the Mendeley software and underwent de-duplication. Literature citations were imported to the Rayyan web application, and abstracts were independently checked by two reviewers (CP and EV), according to the above selection criteria and categorized as included or not included [24]. A third reviewer (ANW) was involved in case of disagreement.

### 2.2. Quality Assessment Strategy and Assessment for Publication Bias

Quality assessment of the selected studies was carried out by two reviewers (KP, CP). ROBINS-I was used to assess risk-of-bias (RoB) [25]. A total score was calculated for each study, according to the number of quality items fulfilled, divided by seven (number of bias domains), yielding a score between 0 and 1, as proposed [25]. We excluded studies judged at high RoB, following the ROBINS-I recommendations [25].

### 2.3. Data Extraction, Synthesis and Reporting

Data were abstracted into a customized data sheet by two authors (GPT and MP), independently. Data included: first author, journal and year of publication, publication type, study design and geographical location of the study, number and age of patients, allergy tests and food allergens considered in the study, the positive predictive value (PPV) of allergy tests and/or the percentage of patients who improved, the outcome confirming culprit allergen, and the main conclusions. The PRISMA guidelines were followed, and AMSTAR 2 was used to provide an accurate and comprehensive summary of the results, as reported in our published protocol [22].

## 3. Results

**Figure 1:**
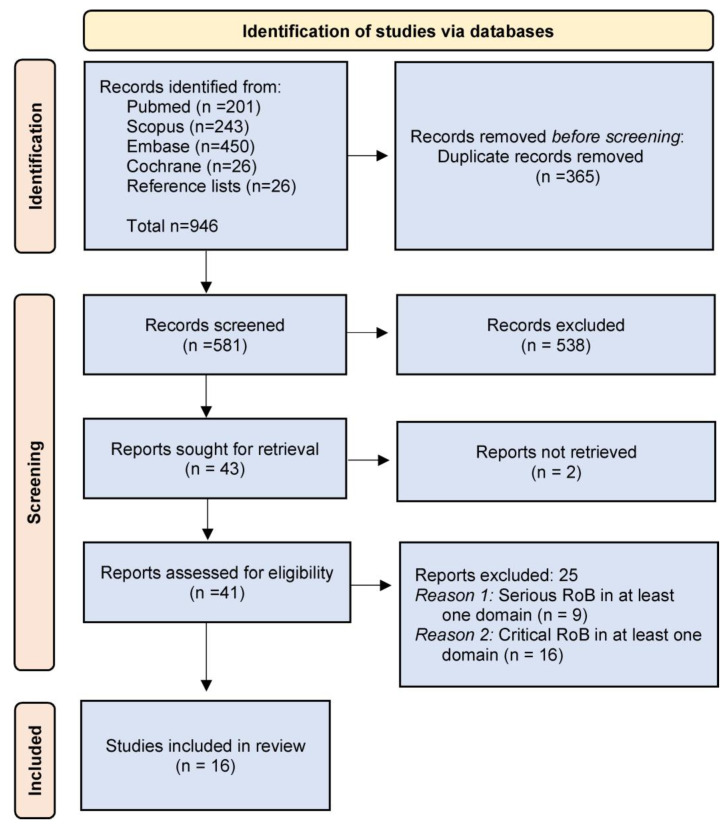
Flow chart.

The evidence search and selection process are presented in Figure 1 (flow chart).

After duplicates removal, 581 unique references were screened for relevance, and 43 were sought for retrieval. Two were not retrieved, although authors were contacted via email. Sixteen studies [26,27,28,29,30,31,32,33,34,35,36,37,38,39,40,41] fulfilling the quality assessment criteria were included in the review; their characteristics are summarized in Table 1. The risk-of-bias ratings of the included studies are shown in Table 2.

No study based on a IgG4-driven diet fulfilled the selection criteria. In one of the studies, prick–prick tests with fresh foods were performed as additional to SPT and APT [32]. In this study, the authors performed prick–prick tests to the same food allergens used for SPT, and their outcome was the detection of more sensitization than SPT and APT, with this skin test method [32]. Although the prick–prick method was not included in our initial protocol, we decided to include the results of the study in our systematic review. The outcomes of another study were also based on prick–prick tests, using raw milk [36].

The characteristics of relevant but not included studies [18,42,43,44,45,46,47,48,49,50,51,52,53,54,55,56,57,58,59,60,61,62,63,64,65] are reported in Table S1 (https://doi.org/10.5281/zenodo.7106535). Based on the ROBINS-I criteria [25], they were excluded due to serious or critical RoB in at least one domain, as shown in Table S2 (https://doi.org/10.5281/zenodo.7106535).

### Study Characteristics

The studies were published between 2002 and 2020, assessing the complete data of 475 EoE patients. These 16 studies were non-randomized: 8 studies were retrospective, while 8 were prospective. The largest study included complete data on 146 children. Biopsies were used for re-evaluation in all studies and as the main criterion for EoE remission in most of the studies.

The positive predictive value (PPV) of allergy tests is reported in Table 3. It is deduced by the percentage of allergy tests that correctly predicted the culprit allergen out of the total number of allergy tests with positive results. The percentage of patients who responded to treatment (similar to tests’ PPV) was calculated by dividing the number of patients who achieved EoE remission after food elimination diets (based on positive allergy tests), out of the total number of patients following such diets. The reviewed studies have offered either, or both, of these data.

The retrieved studies have followed a protocol with a single allergy test (SPT, sIgE or APT) or with a combination of two (SPT+sIgE, SPT+APT) or three (SPT+APT+sIgE). Most studies based only on one allergy test reported PPVs lower than 50%, with the exception of one study that reported a PPV of SPTs as high as 87% (with 92.8% of patients improving) [36]. PPV was better for combined tests; most PPVs were over 50%. However, the results were disappointing in two studies assessing the PPV of both SPT+sIgE, one on children and one on adults [33,39]. The study by Quaglietta et al. extrapolated data from a mixed pediatric population presenting EoE and celiac disease; however, it was clearly mentioned that no child with EoE presented complete symptom or histological remission after following an allergy-test elimination diet [33].

The PPV of the SPT+APT combination was 67.1%, with 65–88.3% of patients presenting symptom amelioration after following a SPT+APT-based elimination diet [28,30,31,34]. The combination of SPT+APT+sIgE was studied only by Dalby et al. [29] reporting symptoms’ improvement in 67% of patients. It can be assumed that the detection of both humoral and cellular sensitization to food allergens and the food elimination of all allergens with positive results from any of the allergy methods offers an increased PPV.

The variety of allergy test methods, allergens tested, ways used to confirm the culprit allergen, periods of food challenge, data chosen to present, and the description of outcomes makes it difficult to extract a safe effectiveness result. A 66.7% case-effectiveness was calculated in the current analysis. The comparison of allergy tests to the confirmed EoE-triggers was not possible due to missing data.

Although most studies were performed in children, age does not seem to affect the PPV, since extreme variations appeared, with fluctuations similar in studies of adults and children.

## 4. Discussion

Exclusion diets drive the remission of EoE symptoms and the recovery of the esophageal mucosa. The prospective of an individualized dietary therapy has led to the targeted elimination diet guided by allergy testing, as an alternative to the elemental and empiric elimination diets. This systematic review has sought to evaluate the efficacy of elimination diets based on allergy testing in EoE patients. It summarized the data of 16 studies, which have not allowed a meta-analysis; however, the evidence in the field was evaluated, and some weaknesses were revealed.

According to a meta-analysis by Arias et al., the effectiveness of an amino-acid-based elemental diet is approximately 90% in both children and adults, and the six-food elimination diets show effectiveness for 72.1% of cases, while the allergy-test-directed elimination is effective in only 45.5% of the cases [66]. We performed the current systematic review focusing only on the effectiveness of elimination diets based on allergy tests and followed a different quality assessment strategy than the systematic review by Arias et al. [66], so a divergence of outcomes was expected.

It appears that allergy-test-driven elimination diets are effective in 66–88.3% of the cases (combining the results of IgE-detection with APT); thus, EoE treatment with allergy-test-targeted diets is not superior to empirical diets. Empirically eliminating foods like milk can be beneficial to a number of EoE patients. The empiric elimination of cow’s milk or dairies is a slightly less-effective strategy than 6-FED, leading respectively to 65% and 56.9% response rates [67,68]. The decision to follow any of these options or alternatively a 4- or 2-food elimination diets is individualized and often selected according to what best fits to a patient’s lifestyle [69].

Given the fact that allergy-test-driven diets do not appear superior to empirical ones, the emerging question is whether the currently available allergen-specific tests remain useful for the diagnosis and treatment of EoE. Besides offering the diagnosis of atopy, they are used to confirm or exclude the suspected diagnosis of concomitant IgE-mediated food allergy and/or respiratory allergy. There are reports of EoE exacerbations during the pollen season [70,71,72,73]. By detecting the culprit allergen that causes allergic rhinitis and/or asthma, pre-seasonal and co-seasonal therapy can be beneficial for respiratory, as well as for EoE symptoms. Nonetheless, the diagnosis of atopy can be a predictive value for the outcome of elimination diets, since it has been reported that atopic patients have been benefited more by a 6-week targeted-diet than non-atopics following a 6-FED [40].

The APT test is one of the allergy testing methods included in this review. Its use for EoE is based on detecting non-IgE, cell-mediated, delayed hypersensitivity reactions [74]. Although the epicutaneously applied patch tests with fresh or dried single-ingredient foods in separate metal chambers have been used in subjects with atopic eczema before, their reliability is not considered high [75]. An effort has been made by the European Task Force on Atopic Dermatitis to standardize the APT protocols, regarding allergen preparation and concentration, the use of Finn chambers, and the criteria for interpretation [76]. In the majority of the included studies in the present review, the methodology of APT has not been described. Therefore, the APT method variability may have biased the results of allergy testing in EoE patients.

Prick tests directly on the esophageal lining is a novel diagnostic method examining the effect of food allergens in EoE [77]. In a study by Warners et al., esophageal prick testing (EPT) was performed after 4 weeks of elemental or empiric diet and was evaluated with endoscopic monitoring for 20 min and repeated endoscopy during the following day [77]. EPT has the advantage of examining the local esophageal response to dietary triggers, which might be completely different from IgE-detection with the usual allergy tests, resembling the phenomenon of local allergic rhinitis. In the present systematic review, the EPT was not considered since it does not yet comprise an established, broadly performed, method.

Food reintroduction after elemental diets and empiric FED have identified cow’s milk as the most common food trigger [21,39,41]. The overall effectiveness of empirical milk-only elimination diets has been reported to be 68.2% [66].The high positive and negative predictive value of the milk IgE-detecting allergy test are clues that milk has different characteristics than other food triggers [26]. In a subset of patients with cow’s-milk-induced EoE, baked milk products are well-tolerated; however, oral provocation is suggested to detect them, since no biomarkers are yet available [78,79].

The activation of T-cells by cow’s milk in EoE seems to be a more systemic phenomenon, affecting the peripheral circulating T-cell repertoire and is not limited to the esophagus. This was the conclusion of a study on the phenotype of peripheral blood mononuclear cells, including patients with milk-induced EoE [10]. Shortly, the authors showed that a Th2-specific T-cell expansion of cells was noticed after stimulation with cow’s milk allergens, in patients with EoE, irrespective of disease activity (EoE-A, subjects following a milk-containing diet) or inactivity (EoE-I, after a milk-elimination diet). Interestingly, the increase of activated (CD3^+^CD4^+^CD154^+^) T-cells had a statistically significant increase only in the EoE-I group. A non-statistically significant increasing trend was noticed in the EoE-A group, reflecting the already stimulated status of T-cells [10]. Expanding this study on other foods might reveal that phenotyping Th2 cells can be a promising EoE biomarker.

According to a preliminary study, ex vivo food antigen stimulation may have the potential to guide elimination diets [80]. Esophageal biopsies were stimulated with food extracts, based on a patient’s clinical history; three of them related to symptom triggering and three might not trigger symptoms. In a 24-hour culture, supernatant-increased levels of IL-5 and/or IL-9 were induced by symptom-related foods [80]; 75% of the food antigen ex vivo challenges matched the patients’ clinical history.

Ongoing research on immunohistochemical biomarkers for diagnostic and therapeutic purposes on EoE has some promising results; i.e., the expression of ALOX15 in the esophageal epithelium may be useful for the diagnosis of EoE in cases not meeting the threshold histological criteria and a low expression of filaggrin with an overexpression of periostin are considered specific for EoE diagnosis [81]. The staining of GATA-3 and T-bet transcriptional regulators may also be useful in the therapeutical monitoring of EoE [82].

## 5. Conclusions

In conclusion, although the use of food-specific IgE-detection and the performance of APT do not seem useful for selecting which food should be eliminated in the frame of EoE treatment, it is a fact that symptoms are exacerbated by different foods in each patient. Promising research on the detection of food-specific markers may help to form and maintain EoE dietary therapies.

## Figures and Tables

**Table 1:** Studies included in the systematic review.

Author, Year	Publication Type	Study Type	Country	Age	Allergy Tests	Food Allergens	Outcome Confirming Culprit Allergens	Conclusion
Terrados, 2020 [26]	Original article	Retrospective	Spain	Children (2.6–15.7 years)	SPT, sIgE	Cow’s milk	Eos reduction in biopsies and symptom remission. Histological responses were defined with a threshold of <15 eos/hpf	Allergy testing-based elimination of milk in children, has high PPV and NPV
Eckmann, 2018 [27]	Original article	Prospective open-label pilot study	United States	Adults	APT	Dairy, egg, peanuts/tree nuts, soy, fish/shellfish, wheat	Symptoms remitted with elimination diet and relapsed after food reintroduction	APT showed poor performance in predicting food triggers in adults with EoE.
Treyster, 2018 [35]	Conference abstract	Retrospective	United States	Children (2–14 years)	SPT, APT	NR	Undefined improvement in biopsies	APT was not useful in identifying the triggering foods.
Ue, 2018 [36]	Conference abstract	Retrospective	United Kingdom	Adults	SPT, P–P, sIgE	Cow’s milk (commercial extract and raw)	Full response in biopsies (<5 eos/hpf)	SPT to raw cow’s milk may have utility in identifying clinically relevant milk sensitization in EoE patients.
Erwin, 2016 [37]	Letter to the editor	Retrospective	Sweden	Children (2–16 years)	sIgE, IgG4	Cow’s milk, egg, wheat, peanut, soy	Eos reduction (<15 eos/hpf) in repeated biopsies	In patients with undetectable milk-sIgE, a milk-elimination diet had better prognosis than in patients with sIgE > 0.35 IU/mL.
Philpott, 2016 [38]	Original article	Prospective observational	Australia	Adults	SPT, sIgE	Almond, cow’s milk, egg white, hazelnut, peanut, shellfish, soy, tuna fish, wheat,	6-FED, followed by food reintroduction and multiples biopsies. The threshold of <15 eos/hpf defined the responses	SPT, sIgE, sIgG, basophil activation test, and patch tests could not predict food triggers.
Lucendo, 2013 [39]	Original article	Prospective	Spain	Adults	SPT, sIgE	Cereal, cow’s milk, egg, fish/shellfish, legumes/peanut, soy	Food elimination-rechallenge and biopsy. Complete response was defined the count of 0–5 and partial the count of 6–14 eos/hpf	PPV is different for the various SPT/sIgE-tested foods. The avoidance of offending foods can maintain disease remission for up to 3 years.
Rodríguez-Sánchez, 2013 [40]	Original article	Prospective observational	Spain	Adolescents and adults	SPT, sIgE	Cereal, cow’s milk, egg, fish, legumes, nuts, shellfish	Eos reduction in biopsies, Wilcoxon rank test to compare mean±SD	The test-directed diet was found more efficient than 6-FED.
Gonsalves, 2012 [41]	Original article	Prospective clinical trial	United States	Adults	SPT	Cow’s milk, egg, fish, peanuts, shellfish, soy, tree nuts, wheat	Food elimination-rechallenge. Wilcoxon rank test to compare peak eos counts before and after diet	In the study’s adult population, SPT provided poor sensitivity in predicting EoE food triggers.
Henderson, 2012 [28]	Original article	Retrospective	United States	Children and young adults (<21 years)	SPT, APT	Cow’s milk, egg, fish/shellfish for 6-FED, soy, peanuts/tree nuts, wheat	Food reintroduction after elimination diet and biopsy, with a threshold of <15 eos/hpf	The skin-testing-driven diet is not as reliable and not as efficient as elemental and 6-FED diets.
Spergel, 2012 [34]	Original article	Retrospective	United States	Children and adolescents (1–18 years)	SPT, sIgE, APT	Cow’s milk, egg, fruits (apples and peaches), grains (rice, wheat, barley, corn, and oat), meats (beef, chicken, turkey, and pork), soy peanut, vegetables (beans, peas, carrots, and potato)	Symptom exacerbation or eos increase (<15 eos/hpf) after food reintroduction or eos decrease (normalization) on specific allergen-free diet	An elimination diet based on SPT/APT has similar results as empiric food removal.
Molina- Infante, 2012 [32]	Letter to the editor	Prospective	Spain	Adults (>18 years)	SPT, P–P, APT	Cow’s milk, egg yolk, fish, fruits (apple, banana, melon, kiwi, peach, strawberry), grains (corn, oat, rice, rye, wheat), meat (beef, chicken, pork), legumes (beans, lentils, peas, soy), peanuts, shrimp, vegetables (potato, tomato)	Symptom and histological (<5 eos/hpf) remission	Prick–prick tests with fresh foods were performed along to SPT. The elimination diet was based on removing every food with at least 1 positive result in any of the skin tests evaluated.
Dalby, 2010 [29]	Original article	Prospective	Denmark	Children and adolescents (1–16 years)	SPT, sIgE, APT	Cow’s milk, hen egg, wheat flour	Symptom exacerbation after the reintroduction of the confirmed allergens	Resolving symptoms after a test-driven elimination diet was not followed by a significant reduction in eos in biopsy.
Quaglietta, 2007 [33]	Original article	Prospective	Italy	Children (<10 years)	SPT, sIgE	Cow’s milk, codfish, egg, wheat	Clinical scores and histological findings (<10 eos/hpf) after diet	Endoscopy was performed in 7 children on an elimination diet based on SPT/sIgE. None had complete symptom remission or histological normalization.
Spergel, 2005 [30]	Original article	Retrospective	United States	Children and adolescents (0.33–20 years)	SPT, APT	Cow’s milk, egg, fruits (apples, pears, and peaches), grains (rice, wheat, barley, corn, rye, and oat), meats (beef, chicken, turkey, and pork), peanut, soy, vegetables (string beans, peas, carrots, squash, potato, and sweet potato)	Βiopsy to confirm remission (<5 eos/hpf defined the responders, 6–15 eos/hpf defined the partial responders) and repeated biopsy after food reintroduction (threshold of >15 eos/hpf for the diagnosis of EoE)	This study distinguishes responders (patients with improvement after a SPT/APT-driven diet) from patients with confirmed causative foods (reintroduction of foods and repeating biopsy after a diet-based increase in eosinophils).
Spergel, 2002 [31]	Original article	Retrospective	United States	Children (2.3–14.3 years)	SPT, APT	Beef, dried egg white, chicken, corn meal, dehydrated potatoes, fish, flour (wheat, oats, barley, rye, and rice), peanut, peas, shellfish, skim milk powder, soy infant formula, tomato	Resolution of the symptoms and improvement in biopsies, with direct comparison of eos/hpf before and after diet. Threshold of >20 eos/hpf was used.	Resolution of the symptoms and improvement in biopsies

Abbreviations: APT, atopy patch test; EoE, eosinophilic esophagitis; Eos, eosinophils; NPV, negative predictive value; NR, not reported; 6-FED, six-food elimination diet; sIgE, specific IgE; SPT, skin prick tests; P–P, prick–prick.

**Table 2:** Quality assessment of the evidence based on ROBINS-I (“risk of bias in non-randomized studies of interventions”).

Author, Year	Risk of Bias	Bias Domains	Total Score
Terrados, 2020 [26]	Moderate	Missing data, classification of interventions	0.71
Eckmann, 2018 [27]	Low	None	1
Treyster, 2018 [35]	Moderate	Missing data, measurement of outcomes	0.71
Ue, 2018 [36]	Moderate	Bias due to confounding, missing data	0.71
Erwin, 2016 [37]	Moderate	Missing data, selective reporting	0.71
Philpott, 2016 [38]	Moderate	Missing data, measurement of outcomes	0.71
Lucendo, 2013 [39]	Low	Missing data	0.85
Rodríguez-Sánchez, 2013 [40]	Low	None	1
Gonsalves, 2012 [41]	Moderate	Missing data, measurement of outcomes	0.71
Henderson, 2012 [28]	Low	Selection bias	0.85
Spergel, 2012 [34]	Low	None	1
Molina-Infante, 2012 [32]	Low	None	1
Dalby, 2010 [29]	Low	None	1
Quaglietta, 2007 [33]	Moderate	Bias due to deviation from intended intervention, missing data	0.71
Spergel, 2005 [30]	Moderate	Bias due to confounding, deviation from intended intervention	0.71
Spergel, 2002 [31]	Moderate	Measurement of outcomes, selective reporting	0.71

**Table 3:** Positive predictive value of allergy tests.

Author, Year	Number of EoE Patients	Number of EoE Patients Who Completed the Protocol	% PPV (Test/Patients)					
			SPT	sIgE	APT	SPT/sIgE	SPT/APT	SPT/APT/sIgE
Terrados, 2020 [26]	31	29	-	-	-	NA/100	-	-
Eckmann, 2018 [27]	8	7	-	-	12.5/25	-	-	-
Treyster, 2018 [35]	45	31	-	-	NA/3	-	-	-
Ue, 2018 [36]	24	23	87/92.8 *	-	-	-	-	-
Erwin, 2016 [37]	27	21	-	NA/38.5	-	-	-	-
Philpott, 2016 [38]	82	23	8.5/10	NA/10	-	NA/5	-	-
Lucendo, 2013 [39]	69	67	21.7/NA	32.9/NA	-	36.2/NA	-	-
Rodríguez-Sánchez, 2013 [40]	30	19	-	-	-	NA/84.2	-	-
Gonsalves, 2012 [41]	50	20	13/NA	-	-	-	-	-
Henderson, 2012 [28]	23	23	12/NA	-	-	-	NA/65	-
Spergel, 2012 [34]	319	14	47/NA	-	44/NA	-	67.1/NA	-
Molina-Infante, 2012 [32]	22	15	-	-	-	-	NA/33.3 *	-
Dalby, 2010 [29]	6	6	-	-	-	-	-	NA/67
Quaglietta, 2007 [33]	17	7	-	-	-	0	-	-
Spergel, 2005 [30]	171	146	-	-	-	-	NA/88.3	-
Spergel, 2002 [31]	26	24	-	-	-	-	NA/75	-

Abbreviations: NA, not available; PPV, positive predictive value; sIgE, specific IgE; SPT, skin prick test; APT, atopy patch test. * Prick-to-prick data are merged.

## Data Availability

The protocol of the systematic review was published [22], https://doi.org/10.1007/s40629-020-00141-7.

## Supplementary Materials

The following supporting information can be downloaded at: https://www.mdpi.com/article/10.3390/jcm11195631/s1, Table S1: Studies not included in the systematic review; Table S2: Quality assessment of the evidence based on ROBINS-I (“risk of bias in non-randomized studies of interventions”) in studies not included in the systematic review.

## Appendix A

**Table A1:** Inclusion and exclusion criteria.

Patients’ characteristics	Patients of any age, with biopsy-confirmed diagnosis of EoE
Interventions of interest	Performance of allergy tests; SPT, sIgE, APT, sIgG4, prick–prick
Comparator	Confirmed food allergenic triggers, causing a histologically confirmed onset or relapse of EoE following their re-introduction after an elimination diet that caused a marked remission of EoE. Similarly confirmed pollen allergens caused a seasonal relapse of EoE. Otherwise, the confirmed diagnosis of allergy is considered the remission of histological and clinical symptoms of EoE, after the exclusion of a single food allergen.
Study designs	Both non-randomized studies of interventions and randomized control trials
Study outcomes	Primary outcome: detection of the relationship between allergy tests and confirmed triggers of EoE Secondary outcomes: effectiveness of allergy tests to be used as positive predictors for the exclusion of food allergens in allergy-driven diets; and the detection of the effect of airborne allergens on the clinical course of EoE
Exclusion criteria	Case reports, case-series, reviews, opinion articles, editorial articles Studies on laboratory animals Studies on oral-induced intolerance Studies without allergy tests, as described in the interventions of interest Studies involving therapeutic procedures, either surgical actions or medication use Studies on tissue IgG4
