# Supplementary material for: Allergy-Test-Based Elimination Diets for the Treatment of Eosinophilic Esophagitis: A Systematic Review of Their Efficacy
Source: J Clin Med. 2022 Sep 24;11(19):5631. doi: 10.3390/jcm11195631 (PMC9572139; doi:10.3390/jcm11195631)
Supplement: Supplementary file 1 [file jcm-11-05631-s001.zip › Table S1.pdf]

Table S1. Studies not included in the systematic review.

Author, year [reference]	Publication type	Study type	Allergy test	Food allergens	Age	Number of EoE patients	Number of EoE patients who completed the protocol	Number of confirmed trigger
Gunderman, 2020 [42]	Conference abstract	Prospective	sIgE, IgG4	Milk, peanut, wheat	Children	66	NR	NR
Dellon, 2019 [48]	Original article	Prospective observational	SPT, IgG4	Egg, milk, peanut, soy, wheat	Adults	24	19	4
Dellon, 2018 [65]	Conference abstract	Prospective	IgG4, Lymphocyte proliferation	Egg, dairy, peanuts, soy, wheat	Adults (16-80 years)	21	14	3*
Schuyler, 2017 [57]	Conference abstract	Prospective	sIgE, IgG4	Cow's milk	Children	9	9	6
Wright, 2016 [18]	Original article	Prospective	IgG4	Dairy, egg, peanut/tree nuts, seafood, soy, wheat	Adults	20	20	11
Eren, 2015 [49]	Conference abstract	Retrospective	SPT, sIgE	NR	Adults (19-71 years)	36	NR	NR
Erwin, 2015 [54]	Conference abstract	Prospective	sIgE	Cow's milk	NA	20	14	9
Kagalwalla, 2015 [50]	Conference abstract	Prospective	SPT, sIgE	Egg, milk, soy, wheat	Children (mean age 9·3 years)	55	55	19
Somoza, 2015 [51]	Conference abstract	Prospective	SPT, sIgE	Legumes	Adults (>14 years)	91	NR	NR
Syrigou, 2015 [52]	Original article	Prospective observational	SPT, sIgE, APT	Beef, chicken, corn, egg, fish, milk, peanut, pork, potato, rice, soy, wheat	Children (0·5-12 years)	35	27	26
Erwin, 2014 [56]	Conference abstract	Prospective	SPT, sIgE	Egg, milk, peanut, soy, wheat and other NR	Children (2-17 years)	10	7	1
Nsouli, 2014 [55]	Conference abstract	Retrospective	SPT, sIgE	Dairy, egg, peach, soy, wheat and other NR	NR	54	NR	82%

Rodríguez-Sánchez, 2014 [58]	Original article	Prospective	SPT, sIgE, APT	Cow's milk, egg, fish, legumes, nuts, shellfish, wheat	Adolescents, adults	45	26	19
Wolf, 2014 [53]	Original article	Retrospective	SPT	Cow's milk, egg yolk, egg white, grains, herbs, meats, fish, fruits, nuts, shellfish, vegetables	Adults	31	NR	NR
Al-Hussaini, 2013 [59]	Original article	Retrospective	SPT, sIgE	Chicken, egg, fish, legumes, milk, shrimp, soy, soybean, peanut, wheat	Children (1-11 years)	18	18	7
Zande, 2013 [60]	Conference abstract	Prospective	SPT, sIgE, APT	Beef, chicken, corn, cow's milk, egg, peanut, pork, potato, rice, soy, wheat	Children	36	30	30
Beser, 2012 [61]	Conference abstract	Prospective	SPT	NR	Children (mean age: 7·24±3·8 years)	42	12	NR
Maggadottir, 2012 [62]	Conference abstract	Retrospective	SPT, APT	Egg, milk, wheat	NR	98	NR	NR
Lleonart, 2011 [63]	Conference abstract	Prospective	SPT, sIgE	Corn, lentils, nuts, peach, rice and other NR	Adults (22-57 years)	11	9	4
Pascual, 2011 [64]	Original article	Prospective	SPT, sIgE, APT	Cow's milk and fractions, beef, chicken, egg yolk, egg white, fish, legumes, nuts	Children (0-14 years)	17	12	8
Antonin-Amerigo, 2010 [43]	Conference abstract	Prospective	SPT, sIgE, APT	Egg, milk, fish, fruits, legumes, nuts, vegetables, wheat	Adults (mean age: 35·7±10·06 years)	17	9	2
Grzywacz, 2010 [44]	Conference abstract	Retrospective	SPT, APT	Chicken, corn, egg, milk, oat, peanut, rice, soy	Children (2-19 years)	36	21	55%
Ramos-Romey, 2009 [45]	Conference abstract	Retrospective	SPT, sIgE, APT	NA	NA	10	10	NA
Spergel, 2007 [46]	Letter to the Editor	Prospective	SPT, APT	Barley, beef, chicken, corn, egg, milk, oat, peanut, potato, rice, soy, wheat	Children	316	74	NR
Simon, 2006 [47]	Original article	Prospective	SPT, sIgE	Rye, wheat	Adults	6	6	0

Abbreviations: APT, atopy patch tests; NA, non-available; NR, not reported; SPT, skin prick test. * Histologic responders.
