# Supplementary material for: Allergy-Test-Based Elimination Diets for the Treatment of Eosinophilic Esophagitis: A Systematic Review of Their Efficacy
Source: J Clin Med. 2022 Sep 24;11(19):5631. doi: 10.3390/jcm11195631 (PMC9572139; doi:10.3390/jcm11195631)
Supplement: Supplementary file 1 [file jcm-11-05631-s001.zip › Table S2.pdf]

Table S2. Quality assessment of the evidence based on ROBINS-I ("Risk Of Bias In Non-randomised Studies of Interventions") in studies not included in the systematic review.

Author, year [reference]	Risk of bias	Domains of bias	Total score
Gunderman, 2020 [42]	Critical	Measurement classification of interventions, measurement of outcomes, missing data, selection of reported result	0.42
Dellon, 2019 [48]	Serious	Missing data, measurement of outcomes	0.71
Dellon, 2018 [65]	Critical	Missing data, measurement of outcomes	0.71
Schuyler, 2017 [57]	Critical	Missing data, measurement of outcomes	0.71
Wright, 2016 [18]	Serious	Missing data, measurement of outcomes	0.71
Eren, 2015 [49]	Critical	Measurement classification of interventions, measurement of outcomes, missing data, selection of reported result	0.42
Erwin, 2015 [54]	Critical	Missing data, measurement of outcomes	0.71
Kagalwalla, 2015 [50]	Critical	Missing data, measurement of outcomes	0.71
Somoza, 2015 [51]	Critical	Measurement classification of interventions, measurement of outcomes, missing data, selection bias, selection of reported result	0.28
Syrigou, 2015 [52]	Critical	Confounding, measurement classification of interventions, measurement of outcomes	0.57
Erwin, 2014 [56]	Serious	Confounding, measurement classification of interventions, missing data, selection bias, selection of reported result	0.28
Nsouli, 2014 [55]	Critical	Measurement classification of interventions, measurement of outcomes, missing data, selection bias, selection of reported result	0.28
Rodríguez-Sánchez, 2014 [58]	Serious	Missing data, Selection of participants	0.71
Wolf, 2014 [53]	Critical	Measurement of outcomes, missing data, selection bias, selection of reported result	0.42
Al-Hussaini, 2013 [59]	Critical	Confounding, deviation from intended interventions, measurement of outcomes, missing data, selection	0.28
Zande, 2013 [60]	Serious	Confounding, measurement classification of interventions, measurement of outcomes, missing data	0.42
Beser, 2012 [61]	Critical	Measurement classification of interventions, measurement of outcomes, missing data, selection bias, selection of reported result	0.28
Maggadottir, 2012 [62]	Critical	Measurement of outcomes, missing data, selection of reported result	0.57
Lleonart, 2011 [63]	Serious	Measurement classification of interventions, missing data	0.71
Pascual, 2011 [64]	Serious	Deviation from intended interventions, missing data	0.71
Antonin-Amerigo, 2010 [43]	Critical	Deviation from intended interventions, missing data, selection of reported results	0.57
Grzywacz, 2010 [44]	Serious	Measurement classification of interventions, measurement of outcomes, missing data, selection bias, selection of reported result	0.28
Ramos-Romey, 2009 [45]	Critical	Missing data, measurement of outcomes	0.71
Spergel, 2007 [46]	Critical	Missing data, measurement of outcomes, selection of participants	0.57
Simon, 2006 [47]	Serious	Missing data, measurement of outcomes	0.71
